# Comments on “Prevalence and risk factors associated with rifampicin-induced cholestasis jaundice among tuberculosis patients in high-incidence setting: a retrospective cohort study”

**DOI:** 10.1016/j.jctube.2026.100618

**Published:** 2026-05-05

**Authors:** Saptanil Das, Archana Dhyani, Rajnish Kumar, Heena Arora

**Affiliations:** a Department of Physiology, Dr. D. Y. Patil Medical College, Hospital and Research Centre, Dr. D. Y. Patil Vidyapeeth (Deemed-to-be-University), Pimpri, Pune 411018 Maharashtra, India; b Faculty of Pharmaceutical Sciences, Graphic Era Hill University, Dehradun, India; c Centre for Promotion of Research, Graphic Era Deemed University, Dehradun, India; d Department of Pharmaceutics, Noida Institute of Engineering & Technology (Pharmacy Institute), Plot No.19, Knowledge Park-II, Greater Noida, India; e Centre of Research Impact and Outcome, Chitkara University, Rajpura 140417, Punjab, India

To the Editor,

Sintusuwan et al. reported clinically important data on rifampicin-induced cholestatic jaundice among patients receiving tuberculosis treatment in a high-burden setting and identified older age and low body mass index as associated factors [1]. The study addresses a relevant safety question and has notable strengths, including a large hospital-based cohort, an effort to exclude alternative causes of jaundice, and reporting of outcomes after rifampicin rechallenge. However, several methodological issues merit clarification before the findings are interpreted as definitive.

First, the description of the source population appears inconsistent. The Methods section states that records were collected between January 2017 and December 2020, whereas the abstract, Results section, and study flow diagram indicate inclusion through November 2022. In addition, the flowchart begins with patients who received rifampicin but later excludes some for no rifampicin use. In observational studies, transparent reporting of eligibility criteria, cohort assembly, and participant flow is fundamental because ambiguity at this stage can affect reproducibility and interpretation [2].

Second, the risk-factor analysis appears vulnerable to selection bias. Although 3,618 patients formed the initial cohort, only 501 were included in the regression analysis, largely because 2,906 lacked HBsAg data. Restricting multivariable analysis to such a selected subgroup may produce estimates that reflect the analytic sample rather than the full rifampicin-exposed population, particularly when no explicit approach to handling missing data or sensitivity analysis is described [2], [3].

Third, the outcome definition and drug-specific attribution would benefit from further methodological justification. Cases were defined by total bilirubin greater than 3 mg/dL or alkaline phosphatase greater than twice the upper limit of normal, yet the reported alkaline phosphatase values suggest that many cases may have had a mixed rather than purely cholestatic pattern. Moreover, because patients were receiving multidrug anti-tuberculosis therapy, attributing liver injury specifically to rifampicin would be strengthened by use of a structured causality framework such as RUCAM or its updated variants [4].

These concerns do not diminish the clinical relevance of the work, but they do suggest that the reported frequency and adjusted odds ratios should be interpreted with caution. Clarification of cohort construction, missing-data handling, and causality assessment would strengthen the validity and generalizability of this otherwise valuable study.

Ethics, Consent to Participate, and Consent to Publish declarations

Not applicable.

Approval of the research protocol by an Institutional Reviewer Board

Not applicable.

Informed Consent

Not applicable.

**Registry and the Registration No. of the study/trial**

Not applicable.

**Animal Studies**

Not applicable.

**Availability of data and materials**

Not applicable.

Statement for studies in humans/animals

No human or animal subjects were involved in this study, as this manuscript is a commentary article. Accordingly, ethical approval and informed consent were not applicable.

## CRediT authorship contribution statement

**Saptanil Das:** Conceptualization, Writing – original draft, Writing – review & editing. **Archana Dhyani:** Conceptualization, Writing – original draft, Writing – review & editing. **Rajnish Kumar:** Writing – original draft, Writing – review & editing. **Heena Arora:** Validation, Writing – review & editing.

## Funding

No funding

## Declaration of competing interest

The authors declare that they have no known competing financial interests or personal relationships that could have appeared to influence the work reported in this paper.
